# Social support and mental health: the mediating role of perceived stress

**DOI:** 10.3389/fpsyg.2024.1330720

**Published:** 2024-02-21

**Authors:** Evelyn F. Acoba

**Affiliations:** ^1^Psychology, Central Luzon State University, Munoz, Philippines; ^2^Department of Psychology, De La Salle University, Manila, Philippines

**Keywords:** social support, perceived stress, mental health, and COVID-19 pandemic, mediation analysis

## Abstract

Social support has been associated with improved mental health; however, the mechanisms underlying this relationship remain unclear. This study aimed to explore whether perceived stress mediate the relationship between social support and positive affect, anxiety, and depression. Drawing from Lazarus and Folkman’s stress and coping theory, the study emphasized the influential role of social support in appraising stressful events. A cross-sectional survey was conducted online among 426 Filipino adults during the peak of the COVID-19 pandemic. Participants completed measures including the Multidimensional Scale of Perceived Social Support (MSPSS), the Perceived Stress Scale-10 (PSS-10), the Positive Affect subscale of PANAS, and the Depression and Anxiety subscales of DASS-21. The hypotheses of the study were tested using mediation analysis. Consistent with the hypotheses, perceived stress significantly mediated the relationship between family and significant other support with positive affect, anxiety, and depression. Family and significant other support decreased perceived stress, increasing positive affect, and decreasing anxiety and depression. On the other hand, perceived stress did not mediate the relationship between friend support and positive affect, anxiety, and depression. Implications and future research directions are discussed.

## Introduction

Social support encompasses feeling loved, valued, and part of a network that offers mutual assistance (Cobb, 1976; Sarason et al., 1987; Wills, 1991). Many studies highlight its protective role in mental health. Yet, the precise ways it impacts mental well-being remain unclear. This gap offers an opportunity for exploration, especially amid the COVID-19 pandemic.

Numerous studies during the pandemic noted increased stress, physical issues, fatigue, loneliness, depression, and anxiety (Brailovskaia et al., 2021; Mansueto et al., 2021; Alhakami et al., 2023). In the Philippines, Tee et al. (2020) and Montano and Acebes (2020) reported widespread mental health concerns during strict quarantines. This crisis reignites interest in understanding how social support boosts mental well-being, given its consistent association with better mental health.

This study delves into how social support benefits mental health amid the COVID-19 pandemic, aiming to uncover insights for future interventions promoting long-term well-being. Anchored in Lazarus and Folkman’s stress and coping theory, the research highlights how social support shapes stress assessment and influences mental health. Understanding these underlying mechanisms is crucial, especially given the current prevalence of mental health issues, and can guide the development of interventions leveraging social support for improved mental wellbeing.

### Social support

Social support, a multidimensional concept, is typically measured in terms of either the structure (number of relationships) or the functions (like informational, instrumental, and emotional) of social networks (Wills, 1991). Perceived support, the perception of available help, often has a more significant impact on mental health than received support (Kessler and McLeod, 1985; Thoits, 1985; Wethington and Kessler, 1986). Research also shows that social support can stem from various sources like family, friends, or pets (Allen et al., 2002; Ford et al., 2007), and can benefit mental and physical health in both stressful and non-stressful times (Cohen and Wills, 1985).

### Social support and mental health

Numerous studies highlight social support’s protective role in mental health. For depression, research underscores the significance of parental support for children, while adults benefit from spouses, family, and friends (Gariepy et al., 2016; Mohd et al., 2019). Low support relates to higher depression, anxiety, and self-harm during pregnancy (Bedaso et al., 2021). Those with weaker support face difficulties in depression recovery and social functioning (Wang et al., 2018). Anxiety among informal carers shows a negative link with perceived support (Priego-Cubero et al., 2023). Social support positively influences well-being in international students and children (Chu et al., 2010; Bender et al., 2019). University students’ well-being and LGB youth’s adjustment tie to perceived support (Watson et al., 2019; Cobo-Rendón et al., 2020). Family support strongly predicts well-being among senior students (Gülaçtı, 2010). Amid COVID-19, higher social support links to lower depression, anxiety, and stress (Grey et al., 2020; Qi et al., 2020; Xiao et al., 2020; Ghafari et al., 2021; Liu et al., 2021).

The Stress and coping theory (Lazarus and Folkman, 1984) provides a framework to understand the pivotal role of social support in mental health. The theory suggests that social support significantly shapes how we perceive and handle stress. It acts as a crucial resource when facing challenges, impacting our stress levels. When we feel supported and equipped to cope, stress tends to diminish. Thus, an event’s perceived stress is not solely inherent; our evaluation, influenced by our perceived social support, greatly shapes how we perceive it. A robust support system can alleviate the overwhelming nature of specific events. Therefore, increasing social support is more likely to decrease perceived stress.

### Perceived stress and mental health

Perceived stress, shaped by how individuals appraise situations and their coping abilities (Lazarus and Folkman, 1984), strongly correlates with mental health outcomes such as anxiety, depression, and harmful behaviors (Liu and Alloy, 2010; Skipworth, 2011; Neese et al., 2013; Zhang et al., 2014; Konstantopoulou et al., 2020; Pieh et al., 2020; Salari et al., 2020; Huang and Zhao, 2021; Puertas-Gonzalez et al., 2023). Epidemiological studies confirm that facing more stressful events and consistently reporting high perceived stress over extended periods are linked to poorer mental health and increased mortality (Epel et al., 2018). The COVID-19 has been a significant stressor impacting mental health, with rising anxiety and depression rates (Courtet et al., 2020; Luo et al., 2020; Pieh et al., 2020; Salari et al., 2020; Huang and Zhao, 2021; Litwin and Levinsky, 2021; Hüfner et al., 2022; Mansueto et al., 2022). Anxiety, a response to stress, shows a strong link to perceived stress, activating similar brain regions (Konstantopoulou et al., 2020; Gijón Puerta et al., 2022). Perceived stress significantly contributes to depression and harmful behaviors like substance abuse (Skipworth, 2011; Neese et al., 2013; Zhang et al., 2014; Reger et al., 2020). Notably, higher positive affect, vital for wellbeing, correlates negatively with perceived stress (Kun, 2013; Eden et al., 2020; Lades et al., 2020; Rackoff and Newman, 2020; Yang and Ma, 2020; Klaiber et al., 2021; Stieger et al., 2021; Zacher and Rudolph, 2021).

### Social support and perceived stress

Previous studies highlight social support’s impact on perceived stress. Özer et al. (2021) linked increased family support to lower stress levels, explaining 11% of stress variance. Ekmen et al. (2021) found family and significant other support significantly reduce stress, with stress partially mediating life satisfaction. McLean et al. (2023) among college students reaffirmed this link: more support meant lower stress. Social support not only directly affects stress but also indirectly influences overall satisfaction by shaping stress perception. This underscores the pivotal role of social support in reducing perceived stress levels.

In summary, empirical evidence consistently indicates a positive link between greater social support and improved mental health outcomes. Further, previous studies have highlighted that social support contributes to mental well-being by diminishing perceived stress. Theoretically rooted in Lazarus and Folkman’s stress and coping theory, social support is recognized as a pivotal factor influencing how individuals perceive and manage stress, consequently impacting mental health. Thus, this study investigates the indirect influence of social support on mental health, specifically through its impact on perceived stress.

This study significantly contributes to advancing our understanding of the intricate interplay between social support, perceived stress, and mental health. By exploring the indirect effect of social support on mental health through perceived stress, it expands upon existing theories and models. Traditionally, the association between social support and mental health has been acknowledged, but this study delves deeper. It elucidates the mechanism by which social support operates, highlighting its role in influencing an individual’s perception and management of stress. The integration of Lazarus and Folkman’s stress and coping theory into this framework offers a theoretical underpinning, illustrating how social support acts as a crucial resource in shaping stress appraisal, subsequently impacting mental health outcomes.

By specifically investigating the pathway through perceived stress, this study bridges gaps in understanding how social support exerts its influence. It augments existing theories by elucidating a more nuanced pathway, shedding light on the nuanced dynamics between social support, stress perception, and mental health outcomes. This deeper understanding can pave the way for more targeted interventions. Understanding the indirect influence of social support on mental health through its impact on perceived stress holds significant clinical relevance. By delving into this relationship, clinicians and practitioners can gain insights into potential intervention strategies. Recognizing the pivotal role of social support in mitigating perceived stress and, consequently, improving mental health outcomes, clinicians can develop targeted approaches.

This comprehension allows for the enhancement of support systems tailored to individuals experiencing high stress levels, thereby potentially reducing the risk of mental health issues. By addressing and bolstering social support perceptions, interventions can be designed to not only alleviate perceived stress but also potentially prevent or ameliorate mental health conditions. This study’s findings may serve as a foundation for developing more effective, nuanced interventions that harness the power of social connections to promote mental wellbeing.

### The current study

Social support has been associated with improved mental health; however, the mechanisms underlying this relationship remain unclear. Hence, this study aimed to investigate whether perceived stress mediate the relationship between social support and mental health outcomes.

This study is based on Lazarus and Folkman’s stress and coping theory, which emphasizes the crucial role of social support as a valuable resource that influences how individuals perceive stressful events. According to this theory, an event is considered stressful when its demands exceed the individual’s available resources, leading to heightened levels of stress. However, if individuals perceive an event as manageable and believe they have the necessary resources to handle it effectively, their experience of stress tends to decrease. A key factor that affects the appraisal process is the presence of strong social support. Having a reliable support network can significantly shape how individuals perceive stressful events. Social support can take various forms, including emotional support, practical assistance, informational guidance, and companionship. When individuals have access to these forms of support, they are more likely to view the event as less stressful. In essence, the appraisal of stressfulness depends not only on the objective nature of the event but also on the individual’s subjective evaluation of their available resources, including social support. A robust support system can contribute to perceiving certain events as less daunting, thereby positively impacting mental health outcomes, and reducing the likelihood of negative psychological consequences.

Building upon Lazarus and Folkman’s stress and coping theory and considering the existing body of research regarding the impact of social support on mental health and its correlation with perceived stress, this study posits the following hypotheses:

1. Social support is positively associated with positive affect and negatively associated with anxiety, and depression symptoms.
2. Social support is negatively associated with perceived stress.
3. Perceived stress is negatively associated with positive affect and positively associated with anxiety and depression symptoms.
4. Perceived stress mediates the association between social support with positive affect, anxiety, and depression.

### Conceptual model

The mediation model is a statistical framework that enables researchers to explore the indirect impact of an independent variable on a dependent variable through a mediator. In this study, the independent variable is social support, the dependent variables comprise mental health outcomes such as positive affect, anxiety, and symptoms of depression, and the proposed mediator is perceived stress.

**FIGURE 1:**
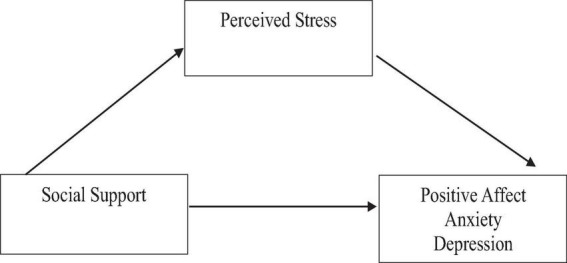
Hypothesized simple mediation model of the relations among social support and mental health and the mediating role of perceived stress.

The proposed mediation model is grounded in Lazarus and Folkman’s stress and coping theory (1984), which emphasizes the crucial role of social support in how individuals evaluate stressful events. Overall, the mediation model posits that social support will have indirect effect on mental health outcomes through a pathway involving perceived stress. Specifically, the model suggests that social support will reduce perceived stress, leading to an increase in positive affect and a decrease in anxiety and depression. Figure 1 presents the schematic presentation of the conceptual model.

## Materials and methods

### Research design

This study utilized a correlational design to explore how perceived stress mediates the relationship between social support and various indicators of mental health, including positive affect, anxiety, and depression. The primary objective was to investigate the potential mechanisms that explain how social support positively influences mental health outcomes.

### Participants

Participants were 426 Filipino adults presently residing in the Philippines. The participants had a mean age of 28.40 years old (age range: 18 - 64 years old, SD = 10.19). Majority of them were females (74.2%), single (80.3%) and were living with their families (88.5%). Most of them were college graduate (48.4%) followed by high school graduates (26.3%) and college students (11.7%). The participants were recruited online, via referral from acquaintances, or through the participants’ referrals themselves. Informed consent was first obtained before answering the research questionnaires.

### Measures

#### Multidimensional Scale of Perceived Social Support (MSPSS)

The MSPSS (Zimet et al., 1988) is a tool designed to assess perceived social support from friends, family, and significant others. It comprises 12 items, with 4 items dedicated to each subscale. The scale has demonstrated robust psychometric properties, including excellent internal consistency (Cronbach’s alpha = 0.95) and strong test-retest reliability (intraclass correlation coefficient [ICC] = 0.91), with kappa values ranging from 0.62 to 0.71. Concurrent validity is evidenced by a negative correlation with perceived stress and a significant positive correlation with rewarding feelings (Wang et al., 2021). In the present study, Cronbach’s alpha for the overall scale is 0.88, and for the subscales, it is 0.91 for significant others, 0.87 for family, and 0.85 for friends. Participants provide responses on a Likert scale ranging from 1 (absolutely disagree) to 7 (absolutely agree), with a higher score indicating greater perceived social support.

#### Perceived Stress Scale-10 (PSS-10)

The PSS-10 (Cohen et al., 1983) was used to assess participants perceived COVID-19 stress levels. The PSS-10 comprises ten items that are rated on a Likert scale ranging from 0 (never) to 4 (very often). The composite score can range from 0 to 40, with higher scores indicating greater perceived stress. The PSS-10 has demonstrated good reliability and validity, with reported Cronbach’s alphas ranging from 0.78 to 0.91 and test-retest reliability coefficients ranging from 0.55 to 0.85 (Cohen et al., 1983; Mitchell et al., 2008). For the present study, a modified version of the PSS-10 was utilized, adapted from the study of Zhang et al. (2021), to reflect perceived stress levels specifically related to the COVID-19 pandemic. The original PSS items were modified to start with the phrase “Since the COVID-19 has occurred,” with an example item being “Since the COVID-19 has occurred, how often have you been upset because of something that happened unexpectedly?”

#### Positive and Negative Affect Schedule (PANAS)

The PANAS (Watson et al., 1988) is composed of two 10-item mood scales that assess both positive and negative affect. For this study, only the positive affect subscale was utilized, as the negative affect was already measured by the DASS-21. Participants were asked to rate the degree to which they have experienced each emotion during a specified period, using a 5-point scale ranging from 1 (not at all) to 5 (very much). The internal consistency reliability of the PANAS scales ranges from.86 to.90 for positive affect and from.84 to.87 for negative affect, indicating good reliability. The Cronbach alpha for the positive affect subscale was 0.89 in this study. Díaz-García et al. (2020) additionally affirmed the suitability of the PANAS when administered online and endorsed its applicability in clinical settings.

#### Depression, Anxiety, and Stress Scales (DASS-21)

The DASS-21 (Lovibond and Lovibond, 1995) are a self-report assessment tool designed to measure levels of anxiety and depression, as well as stress. The items on the DASS-21 are rated on a 4-point scale, with 0 indicating that an item did not apply to the respondent at all and 3 indicating that an item applied to the respondent very much or most of the time. The internal consistency of the DASS-21 scales has been found to be good to excellent in previous research studies, as measured by Cronbach’s alpha (Lovibond and Lovibond, 1995). The validity of the DASS-21 has also been demonstrated consistently. In this study, the stress subscale of the DASS-21 was not included since perceived stress was the predictor variable under consideration. The Cronbach alpha for this study was 0.96. The DASS21 also exhibited strict longitudinal invariance, and the subscales also displayed adequate reliability in the two time points. The results are the first demonstration of longitudinal invariance with a Filipino sample and provide evidence that DASS-21 can be useful in both screening and in monitoring severity of different categories of symptoms over time, particularly among school-based Filipino youth (Simon and Bernardo, 2022).

### Procedure

This study utilized a cross-sectional online survey to collect data between July 21, 2021 and August 5, 2021. The Ethics Committee for Psychological Research was contacted to obtain permission for conducting the survey. Before completing the measures, informed consent was obtained from each participant, and they completed the measures using Google Forms, which ensured anonymity and confidentiality. The survey comprised different measures, including a sociodemographic questionnaire, the Multidimensional Scale of Perceived Social Support (MSPSS), the Perceived Covid-19 Stress Scale-10 (PSS-10), the Positive affect subscale of the Positive and Negative Affect Schedule (PANAS), and the Depression and Anxiety subscales of DASS-21.

### Ethical considerations

The procedures undertaken in this study involving human participants followed the ethical standards set forth by the institutional research committee. Ethics approval was obtained prior to data collection, and participants provided informed consent before completing the measures. The informed consent form contained comprehensive information on the nature and objectives of the study, as well as the requirements expected of the participants. Participants were also assured of the confidentiality and anonymity of their responses. The form indicated the researcher’s contact information in case a respondent experienced lingering discomfort from the survey questions or had further questions related to the study. Furthermore, only the researcher had access to the data obtained from the online surveys, which was stored in a password-protected personal laptop. Data was retained after publication to allow the researcher to address any inquiries about the results and will be deleted a few years after publication.

### Data analysis

Preliminary analyses included checking the assumptions of normality, linearity, and homoscedasticity. Descriptive statistics were calculated for all measures, and the evaluation of the relationships between the variables was performed using the Pearson - r correlation analysis. The bootstrapping method, involving resampling from the data with replacement to create multiple datasets, encompasses the computation of correlations and the examination of correlation coefficient distributions. This technique provides an empirical estimate of the statistical distribution, thereby improving our comprehension of the variability in the targeted statistic. Its application was instrumental in addressing errors stemming from multiple bivariate correlation analyses. The analyses of the mediation effects, were conducted using the IBM SPSS Statistics 25 software and the PROCESS plug-in version 4.0. To test the hypothesis that perceived stress mediated the relationship between social support with positive affect, anxiety, and depression, the bootstrap method was used for the analysis of the effect of mediation (Model 4), with a sample size of 5000. The mediation effect test was considered significant when it did not contain zero under the 95% confidence interval (CI), and the significance level was set at p ≤ 0.05. In the mediation analysis, potential confounders were included as covariates. This involves statistically controlling for the influence of these variables to isolate the direct and indirect effects of the independent variable on the dependent variable through the mediator.

## Results

Table 1 presents the descriptive statistics and bivariate correlations among this study’s variables. Most participants (61.3%) reported a high level of social support from their family, friends, and significant others. In terms of perceived stress, most participants reported moderate levels (78.2%), with a smaller portion indicating high levels (20%), while only 1.9% reported experiencing low stress. Furthermore, most participants reported an average level of positive affect, with an average score of 36.45. However, a significant number of participants (65%) reported experiencing moderate to severe anxiety symptoms, and 49.7% reported moderate to severe depression symptoms.

**TABLE 1:** Descriptive statistics and correlations among study variables.

Variables	Mean	SD	1	2	3	4	5	6
1. Family Support	5.21	1.58	–					
2. Friend Support	5.29	1.47	0.631**	–				
3. SigOther Support	5.29	1.64	0.654**	0.678**	–			
4. Perceived Stress	22.89	4.68	−0.180**	−0.075	−0.119*	–		
5. Positive Affect	36.45	7.94	0.249**	0.201**	0.273**	−0.176** –		
6. Anxiety	15.10	10.34	−0.215**	−0.121*	−0.144**	0.454**	−0.019	–
7. Depression	14.21	10.72	−0.300**	−0.181**	−0.254**	0.505**	−0.179**	0.799**

**p < 0.01

Results of the correlation analysis revealed significant associations between social support and mental health outcomes. Family support displayed a weak yet statistically significant positive correlation with positive affect (r = 0.25, p ≤ 0.01), indicating that heightened family support is linked to increased positive affect. Conversely, family support exhibited weak negative relationships with anxiety (r = −0.22, *p* ≤ 0.01) and depression (r = −0.30, *p* ≤ 0.01), suggesting that greater family support corresponds to reduced levels of anxiety and depression. Furthermore, family support demonstrated a weak negative relationship with perceived stress (r = −0.18, *p* ≤ 0.01), implying that individuals perceiving stronger family support tend to experience lower perceived stress levels.

Similarly, support from significant others exhibited a weak but statistically significant positive relationship with positive affect (r = 0.27, *p* ≤ 0.01) and displayed low negative relationships with anxiety (r = −0.14, *p* ≤ 0.01) and depression (r = −0.25, *p* ≤ 0.01). These results indicate that enhanced support from significant others is associated with increased positive affect and reduced levels of anxiety and depression. Notably, this form of support also showed a low negative relationship with perceived stress (r = −0.12, *p* ≤ 0.01) suggesting that individuals perceiving stronger support from significant other tend to experience lower perceived stress levels.

Further, friend support exhibited a weak positive relationship with positive affect (r = 0.20, *p* ≤ 0.01) and displayed weak negative relationships with anxiety (r = −0.12, *p* ≤ 0.05) and depression (r = −0.18, *p* ≤ 0.05). However, no significant relationship was observed between friend support and perceived stress. These findings reveal the distinct roles of different sources of social support in shaping individuals’ mental health, with family and significant other support appearing to have a more substantial impact compared to friend support.

**FIGURE 2:**
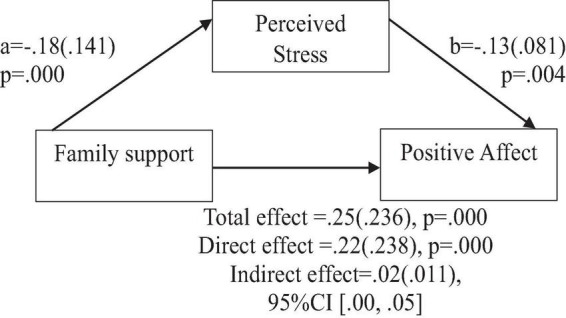
Family support predicts positive affect indirectly through perceived stress. Coefficients are standardized with standard error in parentheses.

**FIGURE 3:**
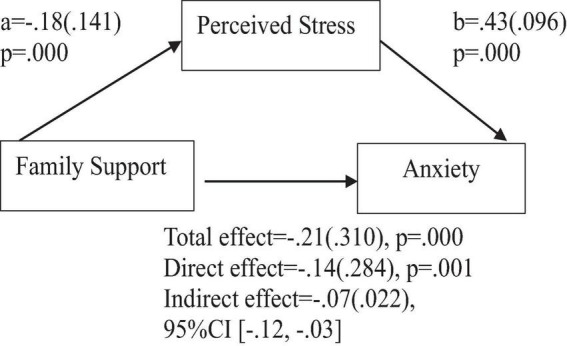
Family support predicts anxiety indirectly through perceived stress. Coefficients are standardized with standard error in parentheses.

**FIGURE 4:**
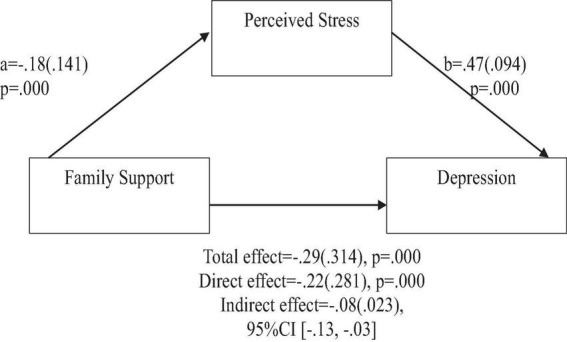
Family support predicts depression indirectly through perceived stress. Coefficients are standardized with standard error in parentheses.

**FIGURE 5:**
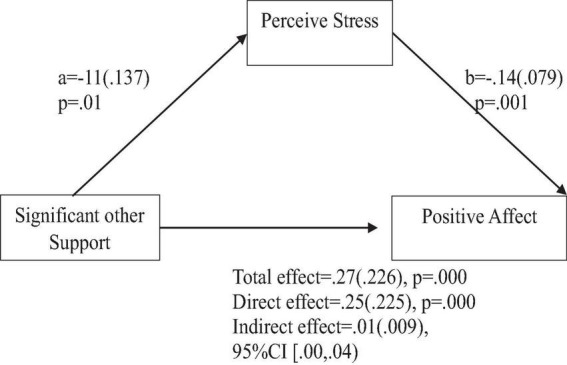
Support from significant other predicts positive affect indirectly through perceived stress. Coefficients are standardized with standard error in parentheses.

**FIGURE 6:**
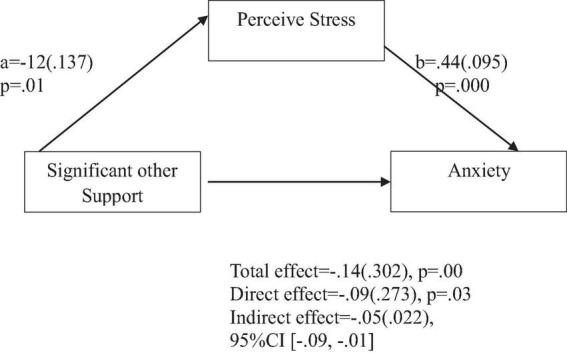
Support from significant other predicts anxiety indirectly through perceived stress. Coefficients are standardized with standard error in parentheses.

**FIGURE 7:**
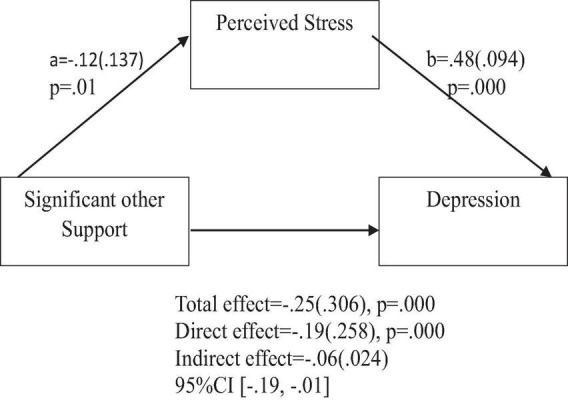
Support from significant other predicts depression indirectly through perceived stress. Coefficients are standardized with standard error in parentheses.

**FIGURE 8:**
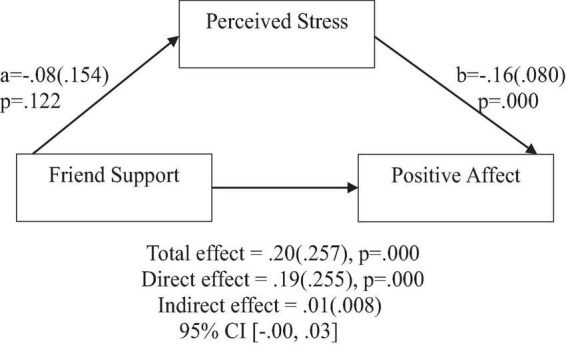
Friend support does not predict positive affect indirectly through perceived stress. Coefficients are standardized with standard error in parentheses.

**FIGURE 9:**
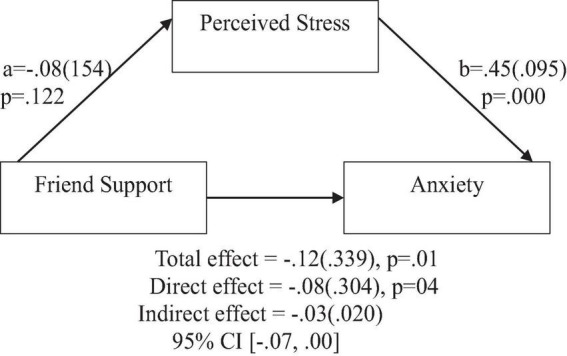
Friend support does not predict anxiety indirectly through perceived stress. Coefficients are standardized with standard error in parentheses.

**FIGURE 10:**
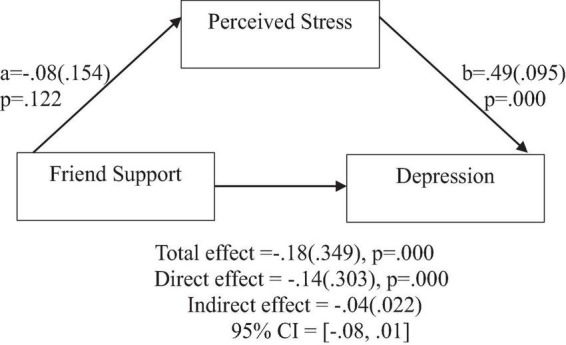
Friend support does not predict depression indirectly through perceived stress. Coefficients are standardized with standard error in parentheses.

To address the question of whether social support predicts positive affect, anxiety, and depression, the findings indicate the following: Based on the total effects, family support demonstrated a significant and positive prediction of positive affect (Figure 2), and a significant negative prediction of anxiety, and depression (Figures 3, 4). Similarly, support from significant other demonstrated a significant and positive prediction of positive affect (Figure 5), and a significant negative prediction of anxiety, and depression (Figures 6, 7). Further, friend support demonstrated a significant and positive prediction of positive affect (Figure 8), and a significant negative prediction of anxiety, and depression (Figures 9, 10).

When considering perceived stress as mediator, the direct effects of family support on positive affect, anxiety, and depression remained significant (Figures 2–4). Similarly, after accounting for perceived stress as mediator, the direct effect of support from significant other on positive affect, anxiety and depression remained significant (Figures 5–7). Furthermore, the direct effects of friend support on positive affect, anxiety, and depression were significant (Figures 8–10).

The study assessed the mediating role of perceived stress on the relationship between family support and positive affect, anxiety, and depression. Figure 2 revealed a significant indirect effect of family support on positive affect through perceived stress [β = 0.02(0.010)], 95% CI [0.00, 0.05]. Family support negatively predicted perceived stress (*a* = −0.18, *p* = 0.000), which in turn negatively predicted positive affect (b = −0.13, *p* = 0.004). Similarly, the study also found a significant indirect effect of family support on anxiety through perceived stress [β = −0.07(0.022), 95% CI (−0.12, −0.03)]. Family support negatively predicted perceived stress (a = −0.18, *p* = 0.000), which in turn positively predicted anxiety (b = 0.43, *p* = 0.000). Furthermore, the study also found a significant indirect effect of family support on depression through perceived stress [β = 0.08(0.023), 95% CI (−0.13, −0.03)]. Family support negatively predicted perceived stress (a = −0.18, *p* = 0.000), which in turn positively predicted depression (b = 0.47, *p* = 0.000).

The study also assessed the mediating role of perceived stress on the relationship between support from significant other and positive affect, anxiety, and depression. Figure 5 revealed a significant indirect effect of support from significant other on positive affect through perceived stress [β = 0.01(0.009), 95% CI (0.00,0.04)]. Support from significant other negatively predicted perceived stress (*a* = −0.11, *p* = 0.01), which in turn negatively predicted positive affect (b = −0.14, *p* = 0.001). Similarly, the study also found a significant indirect effect of support from significant other on anxiety through perceived stress [β = −0.05(0.022), 95% CI (−0.09, −0.01)]. Support from significant other negatively predicted perceived stress (a = −0.12, *p* = 0.01), which in turn positively predicted anxiety (b = 0.44, *p* = 0.000). Furthermore, the study also found a significant indirect effect of support from significant other on depression through perceived stress [β = −0.06(0.023), 95% CI (−0.19, −0.01)]. Significant other support negatively predicted perceived stress (a = −0.12, *p* = 0.01), which in turn positively predicted depression (b = 0.48, *p* = 0.000).

In contrast, friend support was found to have no significant indirect effect through perceived stress on positive affect [β = 0.01(0.008), 95% CI (−0.00,0.03)], anxiety [β = −0.03(0.020), 95% CI (−0.07,0.00)], and depression [β = −0.04(0.022), 95% CI (−0.08,0.01)].

## Discussion

### Social support and mental health outcomes

The current study substantiates the hypothesis that social support, emanating from diverse sources such as family, friends, and significant others, is positively correlated with positive affect and inversely associated with symptoms of anxiety and depression. In essence, heightened perceived social support corresponds to increased positive affect and diminished anxiety and depression symptoms. These findings resonate with prior and more recent research conducted across various cultural and demographic settings (Chu et al., 2010; Gülaçtı, 2010; Gariepy et al., 2016; Wang et al., 2018; Bender et al., 2019; Mohd et al., 2019; Watson et al., 2019; Alshehri et al., 2020; Cobo-Rendón et al., 2020; Grey et al., 2020; Xiao et al., 2020; Özmete and Pak, 2020; Bedaso et al., 2021; Ghafari et al., 2021; Liu et al., 2021; Yildirim and Aziz, 2023; Yıldırım and Green, 2023; Yıldırım et al., 2023; Green et al., 2024).

These compelling findings underscore social support as a pivotal resource stemming from an individual’s social network. This resource manifests in various forms, encompassing emotional, instrumental, and informational support, each catering to distinct needs. Notably, emotional support cultivates a profound sense of love and care, assuring individuals of available assistance when needed. Access to emotional support fosters a secure and accepting environment, facilitating the expression, and processing of emotions. This openness enables individuals to share concerns, vent feelings, and receive empathetic understanding. The impact of emotional support on mental health outcomes is profound, aiding individuals in coping with emotions and bolstering overall well-being. Instrumental support emerges as another influential dimension of social support, offering practical assistance, resources, and guidance in navigating stressors. This facet equips individuals with the tools necessary to effectively confront challenging situations, highlighting the tangible and practical role of social support in mitigating adversity. Additionally, social support extends to informational support, providing valuable information, advice, or feedback. The diverse types and functions of social support thus afford individuals the opportunity to meet various human needs, ultimately contributing to enhanced mental well-being and a reduction in negative mental health consequences.

From a pragmatic perspective, these discoveries hold significant clinical implications. Mental health interventions ought to transcend individual focus, acknowledging the wider social context. Therapeutic strategies that actively engage and harness the support of family, friends, and significant others may demonstrate greater efficacy in fostering positive mental health outcomes. Moreover, interventions could be customized to target forms of support in accordance with individual needs. The integration of these insights into clinical practice shows potential for crafting more holistic and impactful interventions, ultimately playing a role in enhancing mental well-being across varied populations.

### Social support and perceived COVID-19 stress

Consistent with the study’s hypothesis, this research found that social support (from family, friends, and significant others) is negatively associated with perceived stress, indicating that social support decreases perceived stress. These results align with prior studies conducted in diverse cultural and demographic backgrounds (Ekmen et al., 2021; Özer et al., 2021; McLean et al., 2023; Yıldırım and Green, 2023).

Drawing on Lazarus and Folkman’s stress and coping theory, the study illuminates the influential role of social support in shaping our stress perceptions. In the face of challenging events, stress intensifies when we feel overwhelmed and lack coping resources. Conversely, a belief in having adequate support to navigate such events tends to alleviate stress. Social support, manifesting in emotional assistance, practical aid, guidance, and companionship, plays a vital role in this evaluation process. Access to these forms of support enhances our ability to perceive events as less stressful, emphasizing that stressfulness is not solely determined by the event itself but also by our personal evaluations, including the strength of our social support network.

Clinically, these findings underscore the potential of integrating social support interventions into mental health strategies, especially for individuals experiencing heightened stress, such as during a global pandemic. Therapeutic approaches that actively involve and cultivate social support networks emerge as powerful tools in alleviating perceived stress. Mental health practitioners are encouraged to incorporate assessments of social support systems into their evaluations, identifying areas for enhancement and tailoring interventions accordingly.

Healthcare practitioners should acknowledge the stress-alleviating benefits of social support and advise individuals to enhance their social connections, cultivate a sense of community, and promote transparent communication within their social networks. This comprehensive approach recognizes the intricate interplay between individual and social factors, offering a more thorough and effective clinical framework for addressing stress during a global health crisis and beyond.

### Perceived stress, positive affect, anxiety, and depression

Consistent with the study’s hypothesis, the findings revealed a negative association between perceived stress and positive affect, indicating that individuals reporting higher levels of perceived stress exhibited lower levels of positive affect. Furthermore, the study demonstrated that perceived stress was a positive predictor of anxiety and depression, suggesting that heightened levels of perceived stress corresponded to an increased likelihood of experiencing symptoms related to anxiety and depression. These outcomes align with earlier investigations conducted across diverse cultural and demographic contexts (Pieh et al., 2020; Salari et al., 2020; Huang and Zhao, 2021).

The interpretation of these results draws on Lazarus and Folkman’s stress and coping theory. According to this theory, the inverse correlation between perceived stress and positive affect stems from viewing the stressor, in this case, the COVID-19 pandemic, as a threat to one’s well-being, thereby diminishing positive emotional states. Similarly, the positive association between perceived stress and anxiety and depression aligns with the theory’s proposition that unmanaged stress can lead to emotional distress.

Lazarus and Folkman’s stress and coping theory (1984) prove instrumental in comprehending the impact of perceived stress on positive affect, anxiety, and depression in the context of the current study, which focuses on the COVID-19 pandemic as a potential stressor. Recognized as a health emergency, the pandemic embodies characteristics such as harm, life threatening aspects, unpredictability, constraints, trauma, and chronicity, all fulfilling the criteria of a stressor (Wheaton and Montazer, 2017). Additionally, government-mandated mitigating measures add another layer of stress. The nature and demands of the pandemic contribute to prolonged stress experiences, potentially resulting in mental health challenges, as indicated by the study’s findings.

Moreover, the observed decline in positive affect or overall well-being during the pandemic may be attributed to disruptions in activities traditionally associated with enhanced well-being. Activities like outdoor exercising (Reed and Ones, 2006), walking (Hanson and Jones, 2015), and exposure to natural environments (Bowler et al., 2010) are known to promote wellbeing. The pandemic, due to governmental measures aimed at minimizing the virus’s spread, compromises these activities, adversely affecting people’s positive affect.

In a clinical context, these findings emphasize the critical significance of acknowledging and addressing perceived stress as a prominent factor that influences mental health outcomes. Clinicians are encouraged to integrate stress management strategies into their interventions, aiming to alleviate the adverse impact on positive affect and diminish the likelihood of anxiety and depression symptoms. Moreover, a nuanced comprehension of the distinct stressors linked to the pandemic, notably the disruption of activities known to promote well-being, enables targeted interventions to enhance overall mental well-being in the face of challenging circumstances.

Furthermore, as elucidated earlier, healthcare practitioners should recognize and leverage the stress-alleviating benefits inherent in social support. This entails not only acknowledging the role of social connections but actively incorporating strategies to enhance and capitalize on these supportive networks. Such an approach aligns with the multifaceted nature of mental health interventions, integrating both stress management and social support strategies to create a more comprehensive and effective clinical framework.

### Perceived stress as a mediator between the association of social support and mental health outcomes

The outcomes of the present study offer robust support for the hypothesis that perceived stress operates as a pivotal mediator in the relationship between social support from family and significant others and key mental health outcomes, including positive affect, anxiety, and depression. This empirical investigation establishes a clear pathway through which social support exerts its influence, illuminating the role of perceived stress as a critical factor in the enhancement of positive affect and the mitigation of anxiety and depression.

The mediation analyses conducted in this study provide compelling evidence that both family and significant other support possess the potential to elevate positive affect by attenuating perceived stress levels. This suggests that the impact of social support on fostering a positive emotional state operates, at least in part, through the reduction of perceived stress. Furthermore, the findings underscore the effectiveness of family and significant other support in alleviating symptoms of anxiety and depression, emphasizing the role of perceived stress as a key mediator in this relationship. These insights emphasize the nuanced dynamics between social support, perceived stress, and mental health outcomes.

As elucidated earlier, encountering challenging events can induce a sense of overwhelm and resource inadequacy, leading to heightened stress levels. However, the presence of a robust support system, encompassing emotional support, practical assistance, guidance, and companionship, emerges as a buffer against these stressors. Notably, this study highlights the subjective evaluation of stressfulness, suggesting that it is not solely the nature of the events but also the perceived availability of a social support network that shapes our stress experience. Access to social support transforms daunting events into more manageable challenges, thereby significantly contributing to improved mental health outcomes. The study underscores the instrumental role of social support in reshaping individuals’ perceptions of stress and, consequently, their mental well-being.

Finally, the intriguing observation of the absence of an indirect influence of friend support on mental health through perceived stress warrants careful consideration. This observation gains added significance when contextualized within the unique challenges posed by the COVID-19 pandemic. The implementation of stringent mitigation measures, such as social distancing and quarantine, may have substantially restricted opportunities for interpersonal interactions with friends. This altered social landscape likely accounts for the non-significant impact of friend support on mental health through perceived stress. However, it prompts further exploration into alternative mechanisms that may more effectively elucidate the intricate dynamics of how friend support influences mental health outcomes, especially in the context of unprecedented challenges like a global pandemic.

In sum, this study advances our understanding of the nuanced relationships between social support, perceived stress, and mental health outcomes. The identified role of perceived stress as a mediator adds a layer of complexity to our comprehension of how social support operates to shape positive affect and alleviate symptoms of anxiety and depression. These findings carry significant clinical implications, underscoring the importance of incorporating interventions that target perceived stress into mental health strategies, with a particular focus on enhancing social support from family and significant others. Recognizing and fortifying these dynamics can contribute to more effective clinical approaches that address the multifaceted nature of mental health challenges.

## Limitations of the study

The present study’s findings should be interpreted in light of several limitations. Firstly, the cross-sectional design inherently limits the establishment of definitive causality among the study’s variables. Future research endeavors may explore longitudinal designs to ascertain conclusive causality among the variables under investigation. Secondly, the data collected and analyzed relied on self-reported measures, which introduces the possibility of common-method bias. To address this concern, future studies could incorporate additional measures, such as behavioral observations. Thirdly, the online data collection method employed in this study may have implications for the sample characteristics, potentially limiting the generalizability of the findings. To address this issue, future research could explore alternative data collection methods to include individuals who lack internet access. Nevertheless, despite these limitations, the findings of this study significantly contribute to our understanding of human social support and its mechanisms of effects during the Covid-19 pandemic. Moreover, the findings offer valuable empirical evidence that supports the stress and coping theory proposed by Lazarus and Folkman.

## Theoretical implications

The findings of this study carry significant theoretical implications, offering added empirical support for the application of the Stress and Coping Theory (Lazarus and Folkman, 1984) within the context of Filipino adults. Moreover, the study extends this theory by providing evidence for the mechanisms through which social support influences mental health outcomes. By shedding light on the potential mechanisms underlying the positive correlation between social support and favorable mental health outcomes, as well as the inverse relationship with negative mental health outcomes, this research contributes to our understanding of the intricate dynamics at play. Notably, the study highlights perceived stress as a pivotal mediating factor in the connection between familial and significant other support and positive affect, anxiety, and depression. These findings emphasize the critical role of reducing perceived stress as a mechanism through which familial and significant other support can positively impact individuals’ mental well-being. However, it is noteworthy that in this study, perceived stress did not act as a mediating factor in the relationship between friend support and positive affect, anxiety, and depression. This suggests the necessity for further investigation, as alternative mechanisms may provide a more accurate explanation for this association. The absence of a mediating role for perceived stress in the friend support relationship underscores the complexity of social support dynamics and prompts exploration into additional factors contributing to mental health outcomes within the context of friendships.

## Practical implications

From a clinical standpoint, these results hold significant meaning and carry practical implications. Mental health professionals should not only prioritize the assessment of social support networks but also consider the specific mediating role of perceived stress in family and significant other relationships. Recognizing the nuanced dynamics of social support can inform tailored interventions aimed at reducing perceived stress and enhancing positive mental health outcomes. The study’s observation of differing mediation patterns with friend support emphasizes the need for a comprehensive understanding of various social support sources, guiding clinicians to explore diverse mechanisms that influence mental health outcomes in different relational contexts.

### Recommendation

Based on the conclusions derived from this study, several recommendations can be proposed for future research and clinical practice. Firstly, a more comprehensive understanding can be attained by exploring the effects of different types of social support and the moderators influencing these effects. Conducting longitudinal studies is imperative for investigating the lasting impacts of social support on mental health outcomes. Such studies will provide the opportunity to explore more definitive causal relationships among the variables under investigation. In clinical practice, it is vital for mental health professionals to evaluate social support networks and their impact on perceived stress, enabling personalized interventions. Tailoring interventions to capitalize on the distinctive advantages of various sources of social support is crucial considering their differing effects. Delving into the interactions between social support and variables like cultural context or individual differences is essential for a more nuanced understanding. Addressing these recommendations in future research can augment our comprehension of the role of social support in mental health, while in clinical practice, targeted interventions can be developed to foster positive mental health outcomes.

## Data availability statement

The raw data supporting the conclusions of this article will be made available by the authors, without undue reservation.

## Ethics statement

The studies involving humans were approved by the De La Salle University, Manila, Philippines. The studies were conducted in accordance with the local legislation and institutional requirements. The participants provided their written informed consent to participate in this study.

## Author contributions

EA: Conceptualization, Data curation, Formal Analysis, Investigation, Methodology, Writing−original draft, Writing− review and editing.
